# Scientific Scholarship and Impact Factors

**DOI:** 10.3389/fimmu.2013.00079

**Published:** 2013-03-28

**Authors:** Kendall A. Smith

**Affiliations:** ^1^Division of Immunology, Department of Medicine, Weill Medical College, Cornell UniversityNew York, NY, USA

Review articles by experts in a field are highly sought after by traditional for-profit publishers because they serve to draw readers and subscribers to their journal. Also, investigators covet invitations from prestigious journals to write review articles, because they bring widespread respect and admiration to the individual as one of the “Key Opinion Leaders” (KOL). This cachet has evolved into a mutual interdependence of the KOLs and the prestigious journals. This mutual relationship is codified and measured by the Impact Factor of the journal, in that reviews receive many more citations than do other types of articles, including original research articles, because scientists cite them more often than original research articles. A well-researched review article is invaluable because it is assumed that one can rely on a particular reviewer to be correct in the details of a particular subject.

However, there is a “dark side” to the system, in that there is a built-in conflict-of-interest in this interdependence of the publisher, journalists in editorial positions, and the reviewer. In particular, once a prestigious investigator has been invited by a prestigious journal to write a review, the editor-journalists have a vested interest in bringing the article to fruition. The invited authors, on the other hand, have a unique forum to press their particular agendas, to tell the story from their particular viewpoint. We are all aware of this situation, and to the “rewriting of history” by some KOLs. Unfortunately for the readers, especially those new to the field, it may be problematic to trust a particular review author’s hidden agenda. Moreover, some editor-journalists do not send invited-reviews out for others to review to ensure verity. Without the proper editorial quality controls in place, the system is subject to corruption. A very good discussion of the collusion between journalist/editors and KOL reviewers can be found at: http://scholarlykitchen.sspnet.org/2012/04/10/emergence-of-a-citation-cartel

A case in point is a review of interleukin-2 (IL-2) that I was asked to review recently. After reading the manuscript, before agreeing to undertake the review, I told the editor who contacted me that I felt that there were numerous instances where the authors were selective in their choice of references, favoring their own work over those of others, and that I felt that the review needed major revisions. The journalist-editor assured me that he was extremely interested in my opinion, even so. Moreover, he also wanted the review within 7 days, because the journal wanted to include the review in their next issue, which was going to press very soon.

Scholarship in science is made easier than other fields because there is a written record of articles that allows the writer, the journal reviewer, and the reader to verify statements in any article by referring to the original publication. In this particular instance, I called attention to the very first sentence of the manuscript, which stated:

“*Interleukin-2 (IL-2) was first discovered over 35 years ago as an activity present in supernatants of activated human T cells that mediates T cell growth and proliferation* (Morgan et al., 1976).”

This statement belies the fact that IL-2 is a molecule, not simply “an activity in supernatants.” Therefore, I asked the editors and the authors to read this particular report, because this publication, although important in the history of immunology, makes no reference to IL-2 or even to T cell growth factor (TCGF). Instead, this article reports that Lymphocyte Conditioned Media (Ly-CM) can be used to maintain the proliferation of T cells derived from human bone marrow for as long as 13-weeks. By referring to this report as the original description of IL-2, the reviewers ignored more than a decade of reports from numerous investigators of T cell mitogenic activities in leukocyte conditioned media (Gordon and MacLean, 1965; Kasakura and Lowenstein, 1965; Bach et al., 1970; Dutton et al., 1970; Hoffman and Dutton, 1971; Gery and Waksman, 1972; Gery et al., 1972; Schimpl and Wecker, 1972; Plate, 1976). Also, by referring to the Morgan report as the first description of IL-2, the review ignores all of the subsequent work in the interleukin field, which has now shown that there are multiple molecules in leukocyte conditioned media with TCGF activity (e.g., all of the IL-2Rγ-chain cytokines, IL-6, IL-12, and TNFα), and that only by identifying each of the *molecules* is it possible to distinguish one from another.

Lymphocyte Activating Factor (LAF), a macrophage product shown to be mitogenic for T cells, was found to be quite similar to human leukocytic pyrogen (LP), when highly purified LP preparations were tested using antigen-specific proliferation of macrophage-depleted murine lymph node cells (Rosenwasser et al., 1979). The development of the defining TCGF bioassay (Gillis et al., 1978) allowed the functional differentiation of macrophage-derived Lymphocyte Activating Factor (LAF) from lymphocyte -derived TCGF, and showed that LAF is mitogenic for T cells because it markedly enhances their production of TCGF, which was shown to be the actual T cell mitogen in the TCGF bioassay (Smith et al., 1980b,c). Collectively, this work then served as the scientific rationale for the interleukin nomenclature, which was created several years after the 1976 Morgan article. LAF/LP was termed IL-1 because it worked upstream of TCGF, which was given the IL-2 designation. However, additional work was required to identify and characterize the IL-2 molecule as a 15.5 kDa protein with a pI = 8.2 (Smith et al., 1980a, 1983; Robb and Smith, 1981; Robb et al., 1981). Furthermore, these IL-2 molecular characteristics and the defining IL-2 bioassay were instrumental in the successful cloning of the IL-2 cDNA by Taniguchi’s team (Taniguchi et al., 1983). To underscore these points, I referred the editor and the authors to two of my recent reviews, which detail the chronology of contributions to the identification and characterization of what we now know as the IL-2 molecule (Smith, 2012a,b).

The authors and editors chose to ignore my critique and suggestions, and published the article with the original wording and citation. On the day that the article was published, but not before, the editor-journalist wrote:

“*I am certainly not an expert in this field, but I noticed that* (another reviewer) *wrote a review for Nat Rev Immunol in which he called out this paper published in 1976 as the precedent*.”

Thus, as I’ve written before, many for-profit professional journalists perhaps with scientific training, but without the necessary expertise, are making editorial decisions that influence our literature and thus our field. In this regard, it should be realized and stressed to our colleagues and trainees that only journals that are run by scientists as editors and peer reviewers and not by professional journalists can be trusted for the verity and scientific integrity of their publications.

The “Silver Lining” of this dark cloud is that Frontiers can readily distinguish itself from the for-profit closed-access “semi-scientific magazines” by publishing accurate reviews that are vetted and accepted by our scientific peers on our editorial boards. We now have 1638 members of our editorial board, and I am proud of the quality of their work. If we continue to insist on the best scholarship, especially in the reviews we publish, Frontiers will be the “go-to site” by everyone who wants to rely on the verity of the content. The for-profit journalist-editors are handing us the high road on a sliver platter by their shoddy journalism. In our quest to publish “all the science that is fit to read,” we can and should read all of the original references that we cite in our reviews, and our editorial board should insist on accuracy and verity of the citations. This will ensure that our Impact Factor is high.

Pertinent to the point, the recent strategic alliance announced between Frontiers and Nature Publishing Group (NPG), will only serve to underscore the Frontiers commitment to scientific publishing for scientists by scientists. It is to be stressed that Frontiers editors, editorial policies, and management will continue exactly as before the formation of this partnership. Now, this alliance will strengthen and extend the impact of veritable and verifiable publishing by Frontiers. See:

http://www.frontiersin.org/blog/Nature_Publishing_Group_and_Frontiers_form_alliance_to_further_open_science/226; http://www.frontiersin.org/blog/Letter_to_the_Frontiers_editors/228

## References

[B1] Bach F. Alter B. Solliday S. Zoschke D. Janis M. (1970). Lymphocyte reactivity in vitro. II. Soluble reconstituting factor permitting response of purified lymphocytes. Cell. Immunol. 1, 219–227 10.1016/0008-8749(70)90009-2 5523579

[B2] Dutton R. W. McCarthy M. M. Mishell R. I. Raidt D. J. (1970). Cell components in the immune response. IV. Relationships and possible interactions. Cell. Immunol. 1, 196–206 10.1016/0008-8749(70)90007-9 4943430

[B3] Gery I. Gershon R. K. Waksman B. (1972). Potentiation of the T-lymphocyte response to mitogens. I. The responding cell. J. Exp. Med. 136, 128–142 10.1084/jem.136.1.143 5033417 PMC2139184

[B4] Gery I. Waksman B. H. (1972). Potentiation of the T-lymphocyte response to mitogens: the cellular source of potentiating mediators. J. Exp. Med. 136, 143–155 10.1084/jem.136.1.143 5033418 PMC2139186

[B5] Gillis S. Ferm M. M. Ou W. Smith K. A. (1978). T cell growth factor: parameters of production and a quantitative microassay for activity. J. Immunol. 120, 2027–2032 307029

[B6] Gordon J. MacLean L. D. (1965). A lymphocyte-stimulating factor produced in vitro. Nature 208, 795–796 10.1038/208795a0 4223737

[B7] Hoffman M. Dutton R. (1971). Immune response restoration with macrophage culture supernatants. Science 172, 1047–1048 10.1126/science.172.3987.1047 5103320

[B8] Kasakura S. Lowenstein L. (1965). A factor stimulating DNA synthesis derived from the medium of leukocyte cultures. Nature 208, 794–795 10.1038/208794a0 5868897

[B9] Morgan D. A. Ruscetti F. W. Gallo R. (1976). Selective in vitro growth of T lymphocytes from normal human bone marrows. Science 193, 1007–1008 10.1126/science.181845 181845

[B10] Plate J. (1976). Soluble factors substitute for T-T-cell collaboration in the generation of T-killer lymphocytes. Nature 260, 329–331 10.1038/260329a0 1082991

[B11] Robb R. J. Munck A. Smith K. A. (1981). T cell growth factor receptors: quantitation, specificity, and biological relevance. J. Exp. Med. 154, 1455–1474 10.1084/jem.154.5.1455 6975347 PMC2186509

[B12] Robb R. J. Smith K. A. (1981). Heterogeneity of human T-cell growth factor(s) due to variable glycosylation. Mol. Immunol. 18, 1087–1094 10.1016/0161-5890(81)90024-9 6977702

[B13] Rosenwasser L. Dinarello C. Rosenthal A. (1979). Adherent cell function in murine T-lymphocyte antigen recognition IV. Enhancement of murine T-cell antigen recognition by human leukocytic pyrogen. J. Exp. Med. 150, 709–714 10.1084/jem.150.3.709 314491 PMC2185642

[B14] Schimpl A. Wecker E. (1972). Replacement of T cell function by a T cell product. Nature New Biol. 237, 15–17 10.1038/237015a0 4503682

[B15] Smith K. (2012a). Toward a molecular understanding of adaptive immunity: a chronology, Part I. Front. Immunol. 3:369 10.3389/fimmu.2012.00369 23230443 PMC3515840

[B16] Smith K. (2012b). Toward a molecular understanding of adaptive immunity: a chronology, Part II. Front. Immunol. 3:364 10.3389/fimmu.2012.00364 23230441 PMC3515962

[B17] Smith K. Baker P. Gillis S. Ruscetti F. (1980a). Functional and molecular characterization of T cell growth factor. Mol. Immunol. 17, 579–589 10.1016/0161-5890(80)90156-X 6971398

[B18] Smith K. Gilbride K. Favata M. (1980b). Lymphocyte activating factor promotes T cell growth factor production by cloned murine lymphoma cells. Nature 287, 853–855 10.1038/287580a0 6776414

[B19] Smith K. A. Lachman L. B. Oppenheim J. J. Favata M. F. (1980c). The functional relationship of the interleukins. J. Exp. Med. 151, 1551–1556 10.1084/jem.151.1.246 6770028 PMC2185867

[B20] Smith K. A. Favata M. F. Oroszlan S. (1983). Production and characterization of monoclonal antibodies to human interleukin 2: strategy and tactics. J. Immunol. 131, 1808–1815 6352804

[B21] Taniguchi T. Matsui H. Fujita T. Takaoka C. Kashima N. Yoshimoto R. (1983). Structure and expression of a cloned cDNA for human interleukin-2. Nature 302, 305–310 10.1038/302305a0 6403867

